# Molecular docking analysis of a secondary metabolite with the glycoprotein receptors of HSV 1 and HSV 2

**DOI:** 10.6026/97320630015887

**Published:** 2019-12-31

**Authors:** Natarajan Saran, Balaiyah Anandharaj, Giridharan Bupesh, Sakthivel Vasanth, Joseph Pinto Jasmine Beulah, Vellingiri Balachandar

**Affiliations:** 1 Department of Virology, King Institute of Preventive Medicine and Research, Chennai, India; 2 Department of Microbiology, M.R. Government College, Mannargudi, India; 3 Research and Development Wing, Sree Balaji Medical College and Hospital (SBMCH), Bharath University, BIHER, Chrompet, Chennai - 600044, Tamil Nadu, India; 4 Department of Human Genetics and Molecular Biology, Bharathiar University, Coimbatore Tamil Nadu, India

**Keywords:** Herpes simplex virus 1 and 2, glycoprotein, PHYTOL, ADMET properties, drug likeliness

## Abstract

Herpes simplex viruses (HSV) are alpha herpes viruses, which causes life-threatening illness. Therefore, it is of interest to design and develop potential drugs to treat HSV infections. We show the optimal molecular docking properties of a secondary metabolite (3, 7, 11, 15 tetra methyl-2-2-hexadecen-'1-ol) with the glycoprotein receptors of HSV1 and HSV 2 for further consideration.

## Background

Herpes simplex virus 1 and 2 are classified in the family of an alpha herpes virus, which causes mucal lesions in the oral and reproductive organs. Globally 1.6 million new cases of HSV were reported every year, where 22% of adults in USA show antibody for the virus [1,2]. Both HSV 1 and 2 are highly contagious pathogens causing recurrent lesions. Herpes infections are caused by entry in to cells to trigger the envelope film or cell-cell combination [3]. There are four major viral envelope glycoproteins gD, gE, gB, and gH which help in inducing infection in the host by entry and replication in the cells. Glycoprotein D (gD) is the receptor protein which binds activates the other heterodimer such as gH/gL to elicit gB to mediate the fusion of viral and host cellular membranes. Virus entries [4] lead cause recurrent lesions. Therefore, the glycoproteins are key molecular machinery with highly conserved common domains as target for the novel discovery of antiherpes drugs. Ethanolic, chloroform and aqueous extracts of Justicae adothoda leaves were evaluated for the antiviral activity. Among the extracts, the ethanolic extract of Justicae adothoda revealed a promising antiviral activity. Further, the extracts were identified through analytical techniques such as gas chromatography, mass spectra and high-pressure thin layer chromatography. The GCMS chromatogram of ethanolic extract depicts the presence of active compound Phytal.

Phytal is a naturally occurring organic compound belonging to the member of diterpenoids. It is a long chain unsaturated acyclic alcohols, which exhibits wide range of biological activities. It is used as aroma compound with prospective candidate for a wide range of applications in the chemotherapeutics and other biotechnological industry. Phytal exerts many medicinal properties such as antimicrobial, anti-inflammatory, anti nociceptive, anxiolytic, immuno modulating, metabolism modulating, antioxidant, autophagy, and apoptosis inducing activity and cytotoxicity effects. Therefore, it is of interest to design and develop potential drugs to treat HSV infections. We show the optimal molecular docking properties of a secondary metabolite (3, 7, 11, 15 tetra methyl-2-2-hexadecen-'1-ol) with the glycoprotein receptors of HSV1 and HSV 2 for further consideration.

## Methodology

### Preparation and optimization of receptors and ligands:

The 3-dimensional glycoprotein-D, E structures of HSV 1 and 2 were downloaded from the protein data bank (PDB) Research Collaboration for Structural Biology (RCSB) [5] files with PDB IDs 2C36, 3U82, 2GIY and 4MYV). It was optimized using Discovery Studio and then exported into docking tools (MT, Ligplot and Autodock). The receptor protein was analysed using side chain packing, energy minimization and optimization using protein preparation tools. The hydrogen atoms were added, bond orders were assigned and waters molecules were removed from the exterior to 5Å site. The P ionization, optimization, energy minimization was done for the 3D geometry of targeted receptors. The ligands were prepared using ligplot and docking was done using Auto dock. 3-D structures of the ligands were optimized as described else were Banks et al. [6].

The molecular docking framework for HSV-1 protein (PDB: 2KI5) shown was created using Autodock as described elsewhere [7]. Discovery studio was used for visualization of the glycoproteins of HSV 1 and 2 proteins with data corresponding to PDB ID: 2C36, 3U82, 2GIY and 4MYV. The ligand was optimized using ligplot. The van der Waals (vdW) scaling factor fractional incriment was selected as 0.85, 0.25, respectively for ligand atoms. The docking scores were calculated using atomic contact energy, energy transformation and score values [8]. ADME properties of the phytal ligand were calculated using PreADMET/Tox tools [9]. The parameters such as SK logS-pure, SK logS-buffer, SK log P-value and SK logD-value for Cytochrome P450 families CYP-3A4-substrate, CYP-3A4-inhibition, CYP-2D6-substrate, CYP-2D6-inhibition, CYP-2C9-inhibition and CYP-2C19-inhibition, Plasma protein binding, Blood brain wall penetration, Skin permeability, human intestinal absorption, Cellular permeability by Caco2 and cell lines (MDCK) were calculated using PreADMET/Tox tools.

### Molecular properties and drug likeliness prediction: 

Molecular properties and drug likeliness were calculated using molinspiration (https://www.molinspiration.com/). This tool predicts the parameters such as LogP, Total Polar surface Area (TPSA), molecular volume and Rule 5 properties [9]. The log p-value is calculated on the basis of contribution by octanol/water coefficient. TPSA is the sub-atomic polar surface region description of the compound. This parameter determines the drug absorption in intestine, bioavailability, permeability and penetration. The number of rotatable bonds reflects the oral bioavailability of drugs based on the lone pair non-ring bond with non-terminal heavy atoms. The TPSA is calculated as described elsewhere [10]. Drug likeliness of compounds was assessed using the prediction tool Molinspiration.

### Rule of 5 properties:

Atomic descriptors used in Lipinski "Standard Rule of 5 properties" were described elsewhere [2]. It states that most "tranquilize like" particles have logP <= 5, sub-atomic weight <= 500, number of hydrogen bond acceptors <= 10, and number of hydrogen bond contributors <= 5. Atoms disregarding more than one of these guidelines may have issues with bioavailability. The standard is classified as "Rule of 5".

### Number of rotatable bonds (nrotb):

This topological parameter is related to sub-atomic adaptability. It is a descriptor of oral bioavailability of drug like compounds [3]. Rotatable bond is characterized as a single non-ring bond that is limited to non-terminal overwhelming (i.e., non-hydrogen) molecule. Amide C-N bonds are not considered in light of their high rotational vitality barrier.

## Results

**Figure 1:**
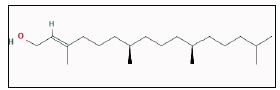
2D structure of the active metabolite 3, 7, 11, 15 tetra methyl-2-2 hexadecen-'1-ol

**Figure 2:**
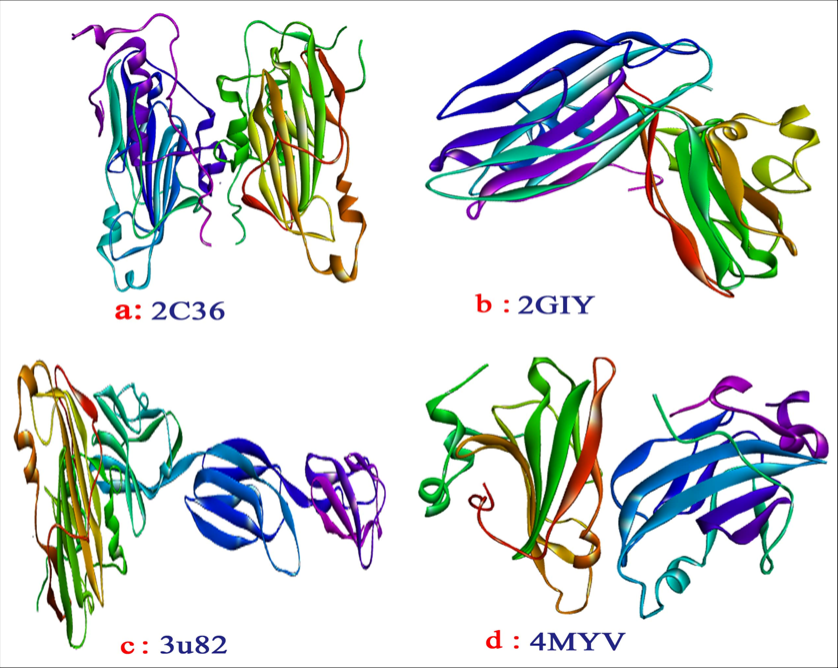
3D structure of envelope glycoprotein from HSV-1 represented using ribbon form and CPK depiction is used for predicted active sites. (a) glyco protein HSV gD with active sites (PDB ID: 2C36); (b) C-terminal domain of the HSV-1 gE ecto domain with active sites (PDB ID: 2GIY); (c) glycoprotein D with active sites (PDB ID: 3U82); (d) gD HSV-2 with active sites (PDB ID: 4MYV).

**Figure 3:**
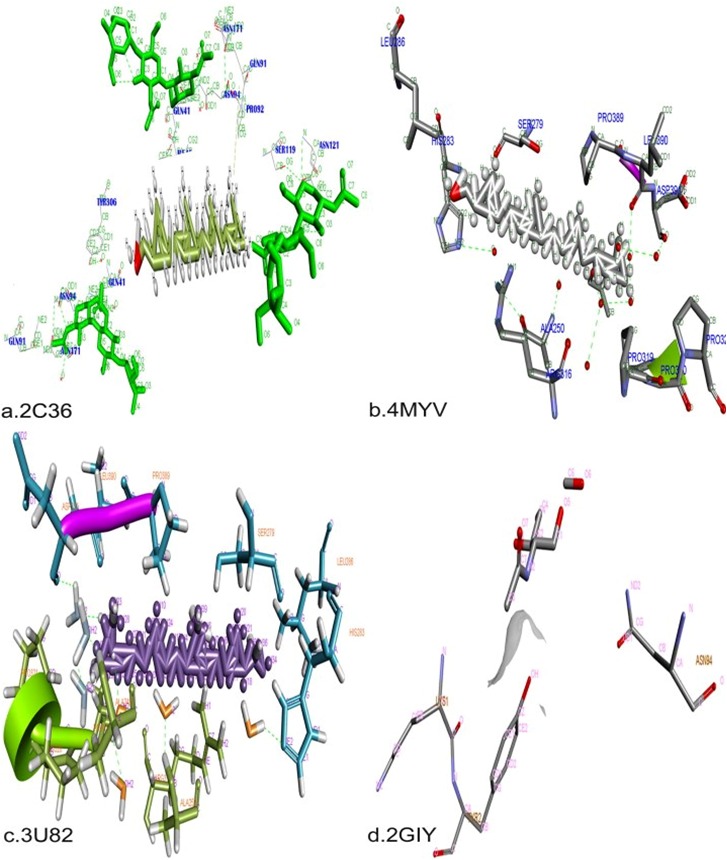
Molecular docking interaction of phytal with the glycoprotein receptors

**Figure 4:**
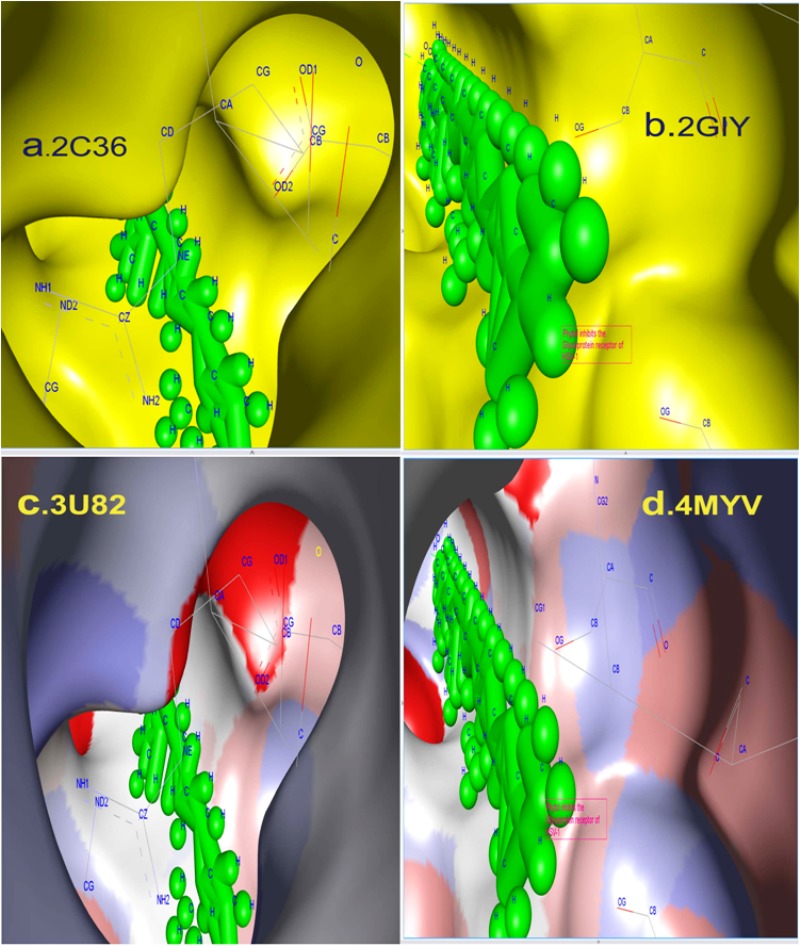
The binding site interaction shown using Discovery Studio

**Figure 5:**
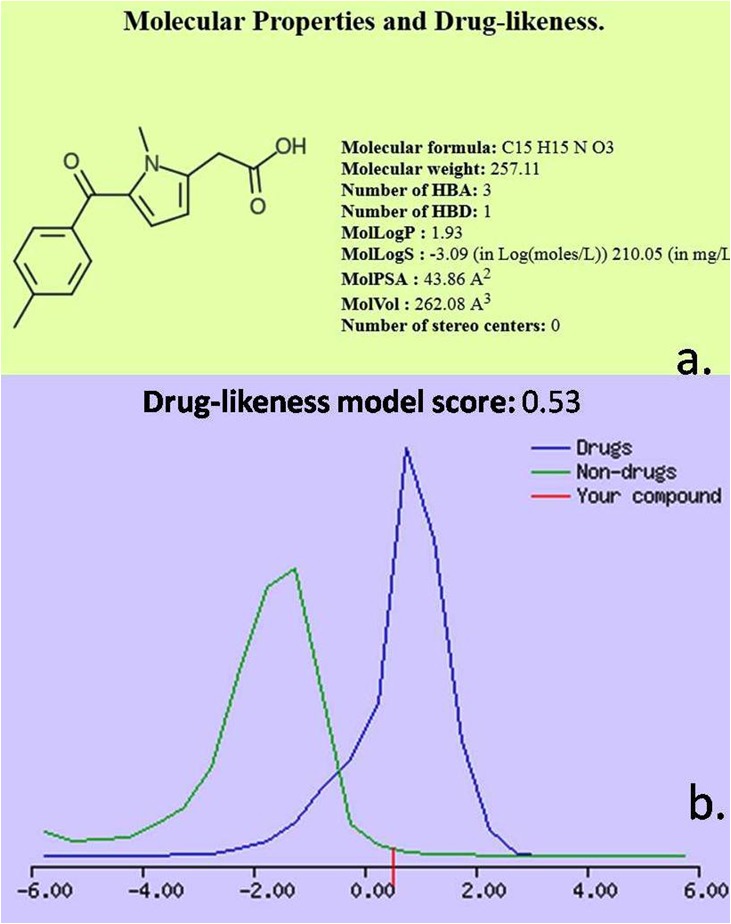
Pharmacokinetic properties from ADMET analysis

The binding of the compound phytal (3,7,11,15 tetra methyl-2-2hexadecen-`1-ol) with the glycoprotein envelope proteins D and E of HSV 1 and 2 is studied in this analysis with ADMET evaluations. Data on the active compounds of Justicia adathoda were known available from Gas chromatography and Mass spectroscopy analysis. The ligand structure is generated using ligplot (Figure 1). The crystal structures of receptors (Figure 2) were optimized using Discovery studio and their binding pockets were identified using molecular tools supported by supercomputing facilities. The docking between phytal and the HSV 1 and 2 glycoprotein receptors gD and gE were completed using AutoDock as shown in Figure 3 and Figure 4 with molecular binding features summarized in Table 1,Table 2 and Table 3. The ADMET properties of the protein-ligand complex are given in Table 4 and Figure 5.

## Discussion

Glycoproteins are the key molecular targets of HSV. There are 11 glycoproteins on the HSV lipid membrane. It is known that 5 among them 5 are linked with cell entry: gB, gC, gD, and the heterodimer gH/gL [3]. HSV infection starts by the effective adherence of the viral envelope glycoprotein-D (gD) to cell surface receptors. These glycoproteins are soluble, truncated ectodomain that exists monomer and in complex state with the ectodomain of its cell receptor HveA. Glycoproteins D have a conceivable restricting locale of receptor named gD receptor. The gD receptor ties with the 3-O-sulfonated heparan sulfate. Suddenly, the structures uncover a V-like immunoglobulin (Ig) crease at the center of gD that is firmly identified with cell attachment atoms and flanked by enormous N-and C-terminal extensions. The receptor adhering fragment of gD, a N-terminal barrette, found adaptable, proposing that a conformational change going with restricting may be a piece of the viral entry mechanism. gD is an objective for both the humoral and cell invulnerable reaction of the host. An HSV-2 subunit vaccine containing a soluble ectodomain of gD is in clinical trials in humans (gD-1 and gD-2 are 85% identical in amino acid sequence).

Three classes of receptors for HSV gD have been depicted. HveA (herpesvirus entry mediator A also called HVEM and TNFRSF14) is a member of the tumor necrosis factor receptor superfamily [11]. It is expressed on activated lymphocytes where it intercedes HSV entry [12]. The cellular function of HveA is to bind the TNF-like ligands. It has been reported that HSV-infected cytotoxic lymphocytes (CTL) rapidly eliminates to other T cells (“fratricide”) and speculated that infection of activated CTLs might provide the virus with a mechanism to evade host immunity. The class-II receptors including HveC immunoglobulin (Ig) also termed as nectin-1 superfamily members with one V-like and two C-like Ig domains. These proteins are functioned as homophilic adhesion molecules on the surface of neuronal and epithelial cells. The class-III receptors are heparan sulfate with 3-O-sulfonated derivatives, which result from D-glucosaminyl-3-O-sulfotransferase-3 alteration of heparan sulfate. The biological consequences of these receptors are still in evaluation. Glycoprotein E is present on the cellular surface of the envelope protein. Glycoproteins E encoded by the gene US8 and have a molecular mass of 80Kda. The glycoprotein E has affinity for the frequent resolution of IgG. It is also known that the binding of IgG inversed the occurrence of glycoprotein I [3].

gD of HSV-1 is a glycoprotein with 369 residues with an N-terminal ectodomain of 316 residues having three N-linked oligosaccharide attachment sites. The receptor has six cysteines residues, which forms 3 disulfide bonds between Cys 66-189, Cys 106-202, and Cys 118-127. Ectodomains of gD truncated residues 285 (gD285) or 275 (gD275) inhibits infection and bind to receptors in vitro 100 times better than a longer form gD306. Hence, we describe the optimal molecular docking properties of a secondary metabolite (3, 7, 11, 15 tetra methyl-2-2-hexadecen-'1 -ol) with the glycoprotein receptors of HSV1 and HSV 2 for further consideration. Glycoprotein D is encoded by the gene US6 and in plays a chief role in antigenic determinant in the HSV-1 viral envelope. The glycoprotein D in the HSV-1 viral envelop undergo frequent mutation for adherence of viral host cell interaction. The mutation in the glycoprotein D (glD) is unable to penetrate the host cell membrane for the establishment of infection in the host.

## Conclusion

We report the optimal molecular docking properties of a secondary metabolite (3, 7, 11, 15 tetra methyl-2-2-hexadecen-'1-ol) with the glycoprotein receptors of HSV 1 and HSV 2 for further consideration.

## Figures and Tables

**Table 1:** Docking of Phytal with Glycoprotein receptors of HSV virus

HSV-1 and 2 receptors with phytal	Score in K/cal	Area	ACE	Ligand Transformation
Glycoprotein HSV-1 gD (PDB.ID: 2C36)	3632	414.9	-64.67	2.34 -1.37 1.38 61.90 60.48 109.22
Glycoprotein HSV-2 gD (PDB.ID: 4MYV)	3874	457.8	-76.89	2.81 -0.18 1.47 2.63 -21.54 11.83
Glycoprotein HSV-1 gE (PDB.ID: 2GIY)	3744	450.9	-57.76	-0.33 0.98 2.36 -34.91 56.82 -13.79
Glycoprotein HSV-1 gD (PDB.ID: 3U82)	3500	392.4	-5.12	1.17 0.20 1.46 11.69 -27.12 -17.63

**Table 2:** Cavities in HSV glyco proteins with active amino acid residues

S. No	HSV glycoprotein	Cavity point	Cavity volume	Catalytic amino acid residues
1	gD HSV-1 PDB.ID:2C36	43.076 69.135 4.848	599	PRO 190,GLU 191,ALA 192, LEU 194, PHE 195, SER 196 PRO 197
2	gE HSV-1 PDB.ID:2GIY	37.725 53.064 137.165	560	VAL 309, TYR 310 and 311, VAL 313, ILE 432, THR 501, SER 502, THR 503, LEU 504
3	gD HSV-1 DB.ID:3U82	5.821 -13.022 -3.323	198	Tyr 116, ALA 121, SER 123, GLU 124,ASP 125, ASN 126, GLY 128, PHE 129, LEU 130
4	gD HSV-2 PDB.ID:4MYV	-0.809 -3.680 25.045	375	SER 7,39; LEU-8, 129, 131; GLU-9, 139;ASP-10; PRO-11, 14,15,73; VAL-76; PHE-12; GLN-13, 142 ILE-19,74,140, LYS-133 THR-60,127,138; MET-66 TYR-128,162; ASN-112

**Table 3:** Bioactivity score of ligands

S. No	MOLINSPIRATION bioactivity	Score
1	GPCR LIGAND	-0.03
2	ION CHANNEL MODULATOR	-0.01
3	KINASE INHIBITOR	-0.3
4	NUCLEAR RECEPTOR LIGAND	0.23
5	PROTEASE INHIBITOR	0.24
6	ENZYME INHIBITOR	0.18

**Table 4:** ADME properties of the Phytal

S. No	ADMET parameters	Value
1	SK logS-pure	-6.82151
2	SK logS-buffer	-6.64222
3	SK logP-value	7.30527
4	SK logD-value	7.30527
5	Skin-Permeability	-0.521653
6	Pure water-solubility-mg-L	0.0444226
7	Plasma Protein Binding	100
8	Pgp-inhibition	Inhibitor
9	MDCK	65.504*
10	HIA	100
11	CYP-3A4-substrate	Substrate
12	CYP-3A4-inhibition	Inhibitor
13	CYP-2D6-substrate	Non
14	CYP-2D6-inhibition	Non
15	CYP-2C9-inhibition	Inhibitor
16	CYP-2C19-inhibition	Inhibitor
17	Caco2	41.2956
18	Buffer solubility-mg-L	0.0671265
19	BBB	19.6582
